# Photosynthesis, Water Status and K^+^/Na^+^ Homeostasis of *Buchoe dactyloides* Responding to Salinity

**DOI:** 10.3390/plants12132459

**Published:** 2023-06-27

**Authors:** Huan Guo, Yannong Cui, Zhen Li, Chunya Nie, Yuefei Xu, Tianming Hu

**Affiliations:** College of Grassland Agriculture, Northwest A&F University, Xianyang 712100, China; huan.guo@nwafu.edu.cn (H.G.); cuiyn@nwafu.edu.cn (Y.C.); lizhen135712@163.com (Z.L.); 17319697319@163.com (C.N.)

**Keywords:** *Buchloe dactyloides*, salt tolerance, photosynthesis, water status, ion homeostasis

## Abstract

Soil salinization is one of the most serious abiotic stresses restricting plant growth. Buffalograss is a C_4_ perennial turfgrass and forage with an excellent resistance to harsh environments. To clarify the adaptative mechanisms of buffalograss in response to salinity, we investigated the effects of NaCl treatments on photosynthesis, water status and K^+^/Na^+^ homeostasis of this species, then analyzed the expression of key genes involved in these processes using the qRT-PCR method. The results showed that NaCl treatments up to 200 mM had no obvious effects on plant growth, photosynthesis and leaf hydrate status, and even substantially stimulated root activity. Furthermore, buffalograss could retain a large amount of Na^+^ in roots to restrict Na^+^ overaccumulation in shoots, and increase leaf K^+^ concentration to maintain a high K^+^/Na^+^ ratio under NaCl stresses. After 50 and 200 mM NaCl treatments, the expressions of several genes related to chlorophyll synthesis, photosynthetic electron transport and CO_2_ assimilation, as well as aquaporin genes (*BdPIPs* and *BdTIPs*) were upregulated. Notably, under NaCl treatments, the increased expression of *BdSOS1*, *BdHKT1* and *BdNHX1* in roots might have helped Na^+^ exclusion by root tips, retrieval from xylem sap and accumulation in root cells, respectively; the upregulation of *BdHAK5* and *BdSKOR* in roots likely enhanced K^+^ uptake and long-distance transport from roots to shoots, respectively. This work finds that buffalograss possesses a strong ability to sustain high photosynthetic capacity, water balance and leaf K^+^/Na^+^ homeostasis under salt stress, and lays a foundation for elucidating the molecular mechanism underlying the salt tolerance of buffalograss.

## 1. Introduction

Soil salinization is an increasingly serious threat to food security and ecological environments worldwide [1]. Salt stress restricts plant growth by affecting a series of physiological processes, such as photosynthetic processes, water uptake and nutrient (especially K^+^) acquisition [2,3]. Photosynthesis, the vital process of primary metabolism, provides energy and organic molecules for plant growth and development [4,5]. However, photosynthesis for numerous plant species is progressively reduced with increasing salinity as a consequence of lessened CO_2_ availability, disturbed chloroplast light energy capture, hindered photosynthetic electron flow and carbon assimilation capacity [6,7]. Photosynthesis is a multi-step and complicated process that comprises several biological pathways [4], and it covers three stages in which many proteins are involved: primary reaction, photosynthetic electron transfer (including photosystem II, cytochrome b6/f and photosystem I complexes) and photophosphorylation (ATP-synthase system), as well as CO_2_ assimilation (such as key enzymes in C_3_ or C_4_ photosynthetic pathways) [8,9,10]. Accordingly, the expression response of genes related to photosynthetic metabolic process is one of the most important reflections for plants coping with salt stress.

The root water uptake is restricted by a diminished water potential because of the presence of high concentrations of salt ions in soil solutions; thus, osmotic stress is also a serious challenge faced by higher plants exposed to saline environments [11,12]. Furthermore, studies have shown that the cell water content of most plant species greatly reduces after only a few hours of salt stress, and the rate of cell elongation and division also significantly slows [13,14,15]. As a consequence, osmotic stress is the primary factor limiting plant growth during the initial stage of salt stress. Acquiring sufficient amounts of water from the saline soil and keeping a higher shoot water content are essential for plants to maintain photosynthetic capacity and cell growth [16,17]. Aquaporins (AQPs) play a pivotal role in regulating water equilibrium inside and outside of the plant cells and water use efficiency (WUE) by specifically mediating the rapid transport of water across cell membranes [18,19]. Plasma membrane intrinsic proteins (*PIPs*) and tonoplast intrinsic proteins (*TIPs*), among the five subfamilies belonging to AQPs, are the most important proteins responsible for water transport because they govern transcellular and intracellular water movement, respectively [20,21], and therefore play vital roles in maintaining the water status of plants grown in saline environments.

The most direct damage of salt stress to plants is ionic toxicity. There are high amounts of salt ions in saline habitats, triggering severe toxicity and nutrient deficiency in plants [22,23]. Na^+^ is the most dominant cation in saline soils, and the overaccumulation of Na^+^ can directly destroy membrane systems, damage cellular organelles and impair photosynthesis [24]. Specifically, it competes with K^+^ binding sites on cellular transporters and channels, causing K^+^ deficiency in cells, which in turn affects protein biosynthesis and enzyme activation [25,26]. The ability of plants to resist Na^+^ toxicity mainly depends on whether they can maintain a high K^+^/Na^+^ ratio in the cytoplasm [11,27]. Additionally, K^+^/Na^+^ homeostasis is an important aspect to maintain the intracellular environment under saline conditions, thereby influencing plant photosynthetic efficiency and water balance. A direct strategy for plants to cope with excessive Na^+^ accumulation and a K^+^ deficit is to regulate the expression of several genes encoding membrane transporters or channels associated with Na^+^ and/or K^+^ uptake, translocation or compartmentalization [28]. For example, the expression of plasma membrane Na^+^/H^+^ antiporter gene *SOS1* in root tips functions in the efflux of Na^+^ from the roots, and the expression of the tonoplast Na^+^/H^+^ antiporter gene *NHX1* in roots/shoots helps with the sequestration of Na^+^ in vacuoles to avoid disturbance to cell metabolisms [3,29,30]; the expression of the high-affinity K^+^ transporter genes *HAKs* and inward-rectifier K^+^ channel gene *AKT1* in roots facilitates the K^+^ uptake [26,31,32]. Therefore, the genes related to Na^+^ and K^+^ transport are also closely associated with the salt tolerance of plants.

Buffalograss (*Buchloe dactyloides* (Nutt.) Engelm.), a C_4_ perennial turfgrass and forage belonging to Poaceae, is native to northwestern America and is now widely distributed in arid and semi-arid regions around the world [33,34,35]. It is the preferred grass for the establishment of landscape green land, the governance of desert saline-alkali land and slope protection due to its outstanding tolerance to various abiotic stresses, rapid reproduction, dense growing habit as well as long lifetime [36,37,38]. Meanwhile, this species is also an attractive year-round forage grass because of its high palatability, rich nutrition and strong grazing resistance [39]. Researchers have found that buffalograss could well-tolerate a certain salinity (up to a 100 mM salt concentration) [33,40]. However, regarding the physiological mechanism of salt tolerance in this species, particularly the photosynthetic responses, hydration status and ion accumulation characteristics under salt treatments have not been intensively documented.

The objective of this study was to characterize the physiological responses of buffalograss to salinity by measuring parameters related to photosynthesis, water conservation and K^+^/Na^+^ homeostasis under treatments with different concentrations of NaCl. Furthermore, the expression patterns of some key genes involved in photosynthesis, encoding *PIP*/*TIP* aquaporins and K^+^/Na^+^ transporters/channels under NaCl treatments were analyzed.

## 2. Results

### 2.1. Buffalograss Exhibited a Strong Tolerance to Salinity

**Figure 1:**
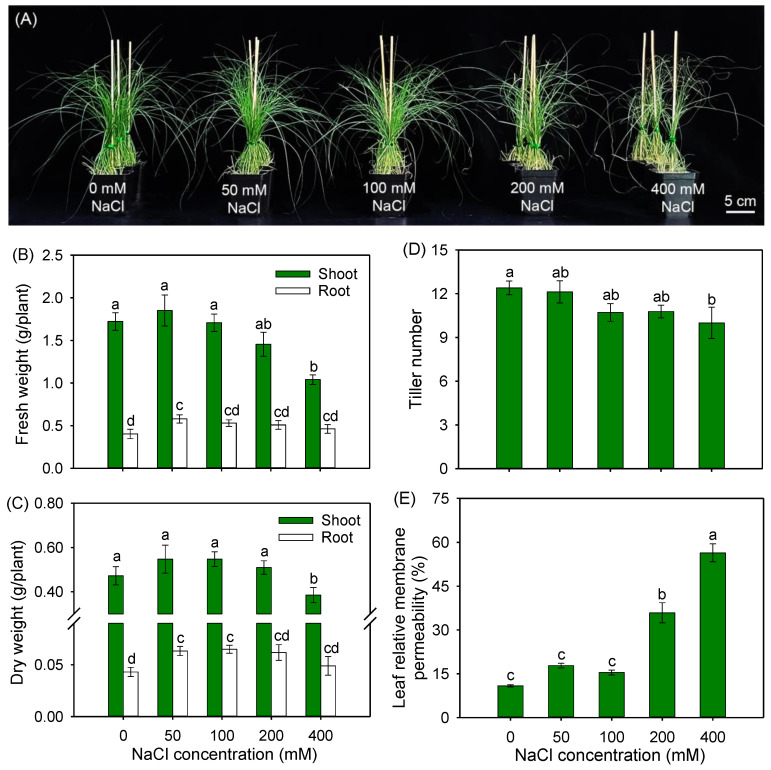
The growth responses (**A**), fresh weight (FW) (**B**), dry weight (DW) (**C**), tiller number (**D**) and leaf relative membrane permeability (RMP) (**E**) of *B. dactyloides* seedlings under different NaCl treatments for 8 days. Values in (**B**–**E**) are means ± SE (*n* = 6) and bars indicate SE. Columns with different letters indicate significant differences at *p* < 0.05 (Duncan test).

After treatment with 50 and 100 mM NaCl for 8 days, the seedlings remained vigorous in growth (Figure 1A). When the salinity increased to 200 mM, the leaf tips of seedlings appeared slightly wilting, and under higher salinity (400 mM NaCl), the leaves of plants were withered and grew weakly (Figure 1A). To further confirm the above observations, plant biomass, tiller number and leaf relative membrane permeability (RMP) were measured. The data showed that the addition of 50, 100 and 200 mM NaCl had no significant effects on plant biomass, tiller number and leaf RMP (except for the RMP exposed to 200 mM NaCl, which was higher than that for the control plants) (Figure 1B–E). It is worth noting that, under 50 and 100 mM NaCl treatments, the root fresh weight (FW) and dry weight (DW) significantly increased by 44 and 47%, as well as 32 and 51%, respectively, in comparison with those under the control condition (Figure 1B,C). A total of 400 mM NaCl treatment resulted in an obvious decrease in shoot biomass and tiller number, and a substantial increase in leaf RMP when compared with the control (Figure 1B–E).

### 2.2. The Photosynthetic Responses of Buffalograss Exposed to Salinity

**Figure 2:**
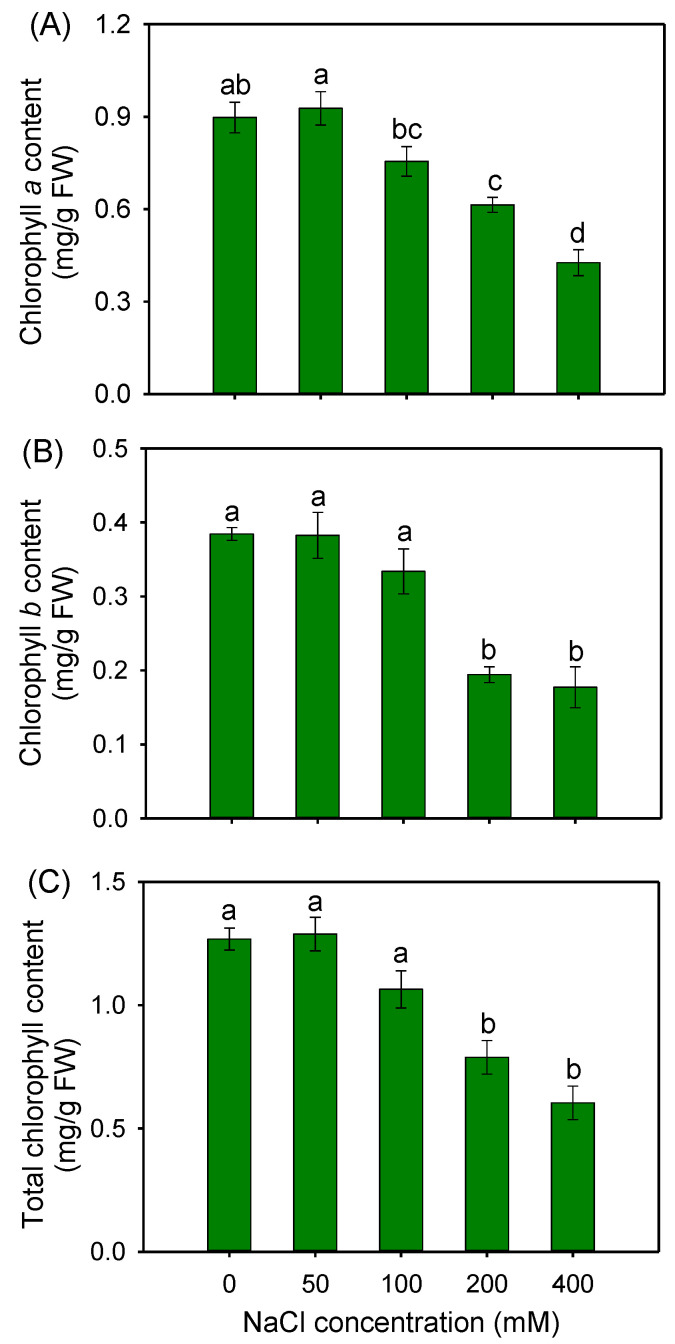
Chlorophyll *a* (**A**), chlorophyll *b* (**B**) and total chlorophyll (**C**) contents of *B. dactyloides* seedlings under different NaCl treatments for 8 days. Values are means ± SE (*n* = 6) and bars indicate SE. Columns with different letters indicate significant differences at *p* < 0.05 (Duncan test).

To investigate the photosynthetic capacity of buffalograss seedlings under saline conditions, the content of chlorophylls and photosynthetic physiological indexes were measured. The results showed that after 50 and 100 mM NaCl treatments, the content of chlorophyll a, chlorophyll b and total chlorophyll in leaves maintained stability compared to that of control plants (Figure 2), suggesting that these two salinities had no significant effect on the chlorophyll synthesis of buffalograss. However, 200 and 400 mM NaCl treatments resulted in an obviously decreased content of chlorophylls (Figure 2).

**Figure 3:**
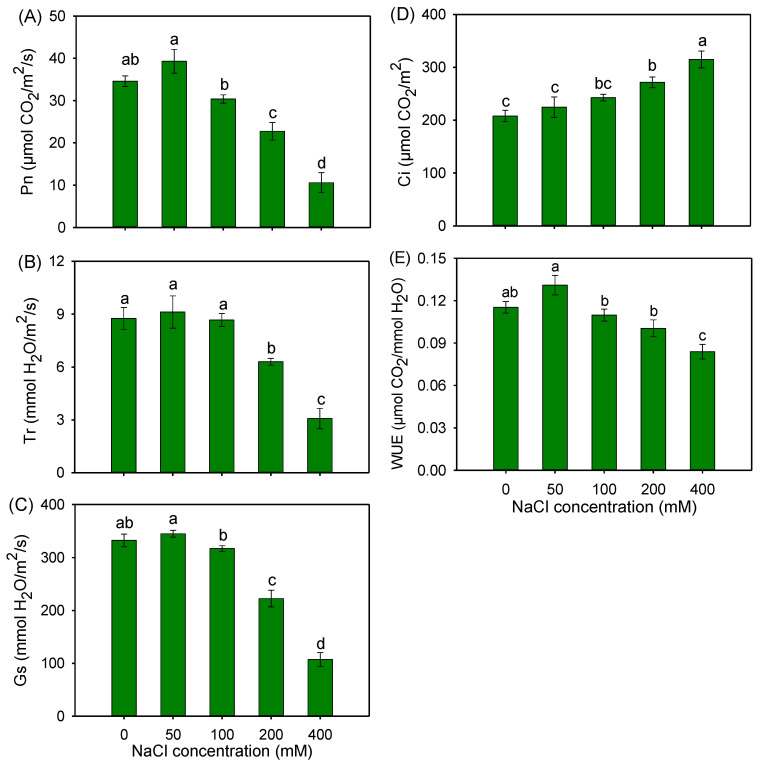
Net photosynthesis rate (Pn) (**A**), transpiration rate (Tr) (**B**), stomatal conductance (Gs) (**C**), intercellular CO_2_ concentration (Ci) (**D**) and water use efficiency (WUE) (**E**) of *B. dactyloides* seedlings under different NaCl treatments for 8 days. Values are means ± SE (*n* = 6) and bars indicate SE. Columns with different letters indicate significant differences at *p* < 0.05 (Duncan test).

The net photosynthetic rate (Pn), transpiration rate (Tr), stomatal conductance (Gs) and intercellular CO_2_ concentration (Ci) of buffalograss seedlings under 50 and 100 mM NaCl were comparable to those under the control condition (Figure 3A–D). However, 200 and 400 mM NaCl treatments significantly reduced Pn, Tr and Gs (Figure 3A–C), but increased Ci (Figure 3D). Notably, buffalograss could maintain a stable leaf water use efficiency (WUE) when plants were exposed to 50–200 mM NaCl treatments (Figure 3E).

### 2.3. The Leaf Water Status and Root Activity of Buffalograss Seedlings Exposed to Salinity

**Figure 4:**
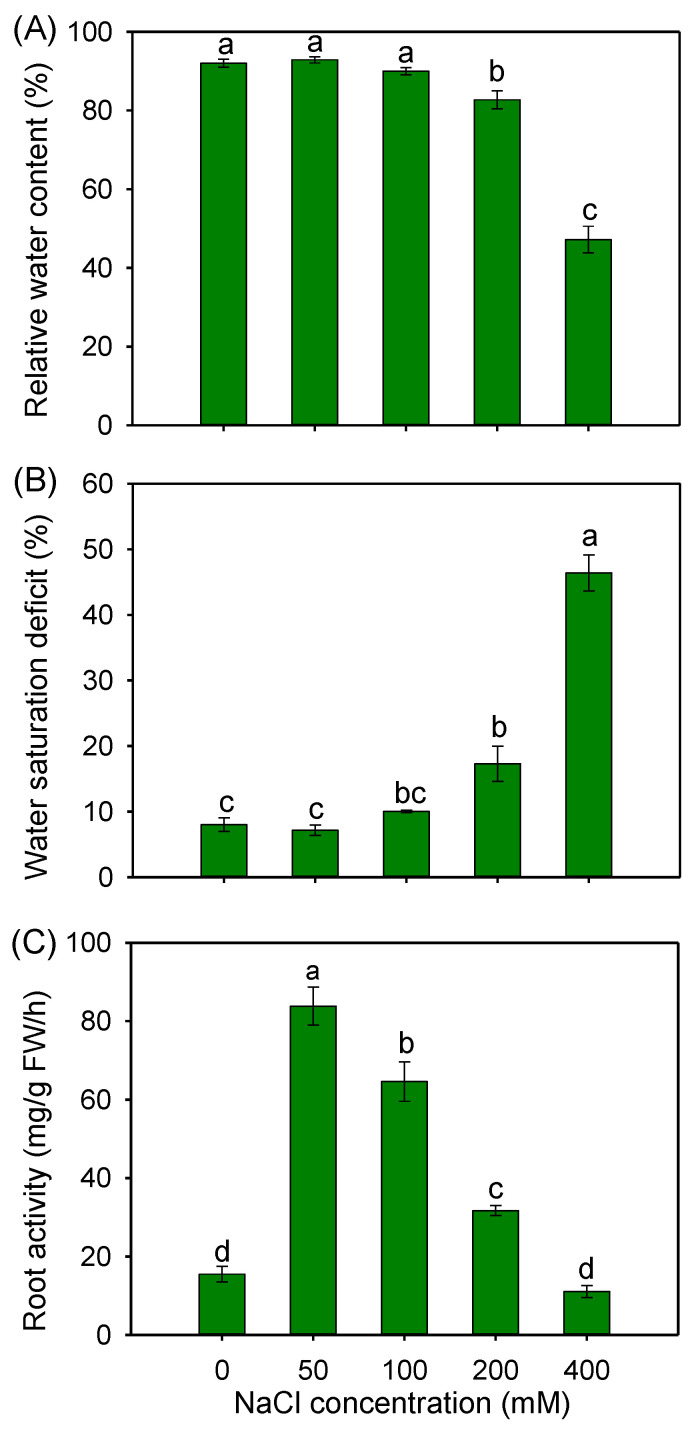
Leaf relative water content (**A**), leaf water saturation deficit (**B**) and root activity (**C**) in *B. dactyloides* seedlings under different NaCl treatments for 8 days. Values are means ± SE (*n* = 6) and bars indicate SE. Columns with different letters indicate significant differences at *p* < 0.05 (Duncan test).

To evaluate the water status of buffalograss under NaCl treatments, the leaf hydration and root activity were measured. As shown in Figure 4A,B, 50 and 100 mM NaCl had no significant effect on the leaf relative water content (RWC) and water saturation deficit (WSD). When treated with 200 and 400 mM NaCl, the RWCs were significantly decreased by 10.1% and 48.7%, respectively, and the WSDs were correspondingly significantly increased by 116.2% and 480.2%, respectively, compared with the those of the control condition (Figure 4A,B). Unexpectedly, in comparison with the control, 50, 100 and 200 mM NaCl treatments significantly increased the root activity by 5.4-, 4.2- and 2.0-fold, respectively (Figure 4C).

### 2.4. The K^+^/Na^+^ Homeostasis in Buffalograss Seedlings Exposed to Salinity

**Figure 5:**
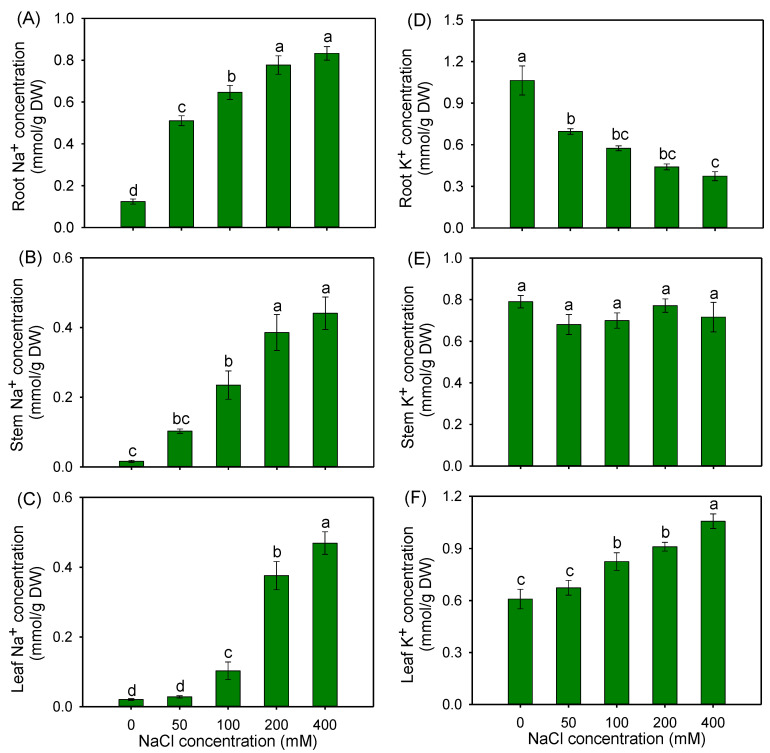
The Na^+^ (**A**–**C**) and K^+^ (**D**–**F**) concentrations in roots, stems and leaves of *B. dactyloides* seedlings under different NaCl treatments for 8 days. Values are means ± SE (*n* = 6) and bars indicate SE. Columns with different letters indicate significant differences at *p* < 0.05 (Duncan test).

With the increase in the external NaCl concentration, the amounts of Na^+^ accumulated in different tissues of buffalograss seedlings were gradually elevated (Figure 5A–C). It was noticed that Na^+^ contents in stems and leaves were lower than those in roots under all salt treatments (Figure 5A–C), indicating that this species could efficiently restrict the long-distance transport of Na^+^ from roots to shoots. Although the K^+^ accumulations in roots were significantly reduced with the increase in the external NaCl concentration, this parameter remained stable in stems, and unexpectedly, it gradually increased in leaves (Figure 5D–F). The leaf K^+^ concentrations in the presence of 100, 200 and 400 mM NaCl were increased by 35.5, 49.7 and 73.6% compared with those under the control condition, respectively (Figure 5F).

**Figure 6:**
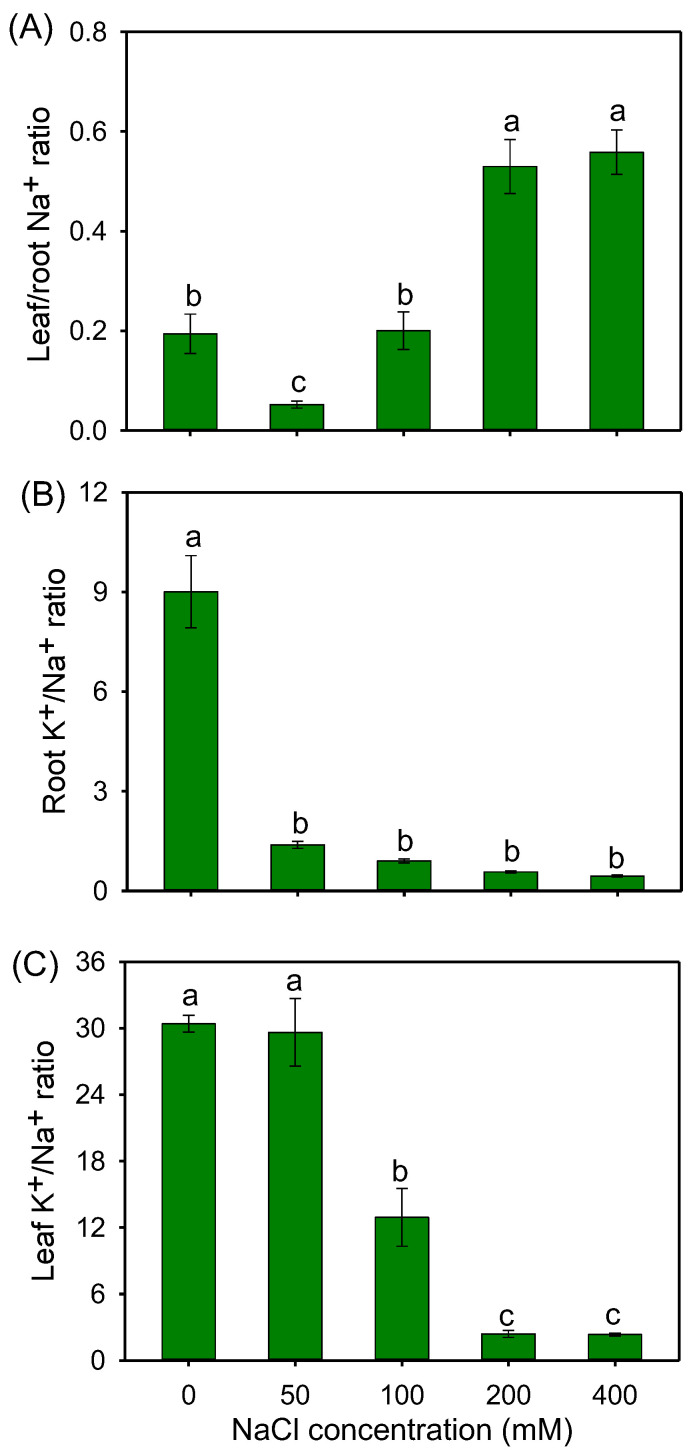
The leaf/root Na^+^ ratio (**A**), root (**B**) and leaf (**C**) K^+^/Na^+^ ratio in *B. dactyloides* seedlings under different NaCl treatments for 8 days. Values are means ± SE (*n* = 6) and bars indicate SE. Columns with different letters indicate significant differences at *p* < 0.05 (Duncan test).

Compared with the control, the leaf/root Na^+^ ratio was significantly decreased by 70.4% under 50 mM NaCl, but sharply increased under 200 and 400 mM NaCl treatments (Figure 6A). In comparison with the control, all the NaCl treatments significantly decreased the K^+^/Na^+^ ratio in the roots (Figure 6B), while in the leaves, the K^+^/Na^+^ ratio under 100–400 mM NaCl treatments significantly declined, but it was maintained at the control level under a 50 mM NaCl treatment (Figure 6C). In addition, the K^+^/Na^+^ ratio in leaves under all salt treatments was greater than one, and was obviously higher than the root K^+^/Na^+^ ratio (Figure 6B,C).

### 2.5. Effects of Salinity on Expression of Genes Related to Photosynthesis in Buffalograss

**Figure 7:**
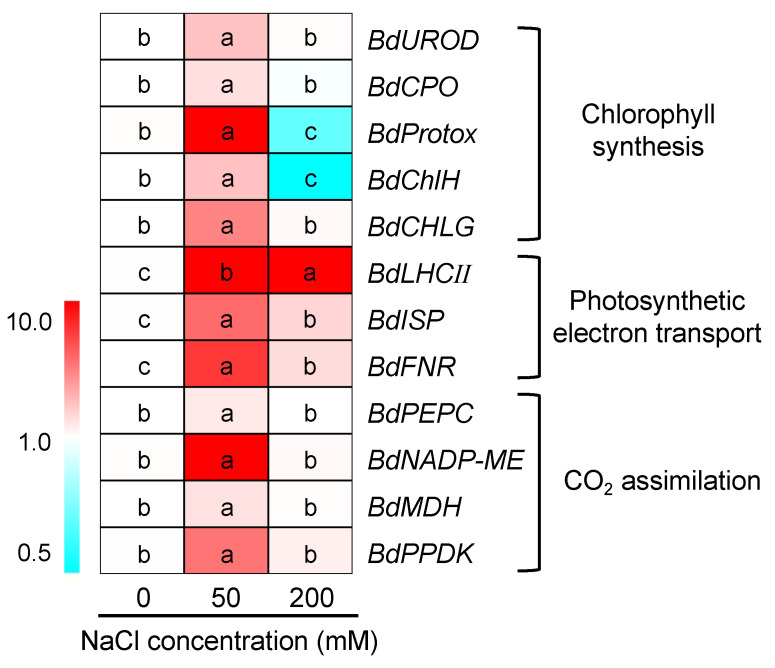
The expression of genes related to photosynthesis in *B. dactyloides* under 0, 50 and 200 mM NaCl for 6 h. Expression of the *B. dactyloides ACTIN* gene was used as an internal control for normalization. Different lowercase letters within each gene indicate significant differences (*p* < 0.05, Duncan test). UROD: uroporphyrinogen decarboxylase, CPO: coproporphyrinogen-III oxidase, Protox: protoporphyrinogen oxidase, ChlH: magnesium chelatase H subunit, CHLG: chlorophyll synthase, LHCII: LHCII type I chlorophyll a-b binding protein, ISP: cytochrome b6/f complex iron-sulfur subunit, FNR: ferrdoxin-NADP^+^ reductase, PEPC: phosphoenolpyruvate carboxylase, NADP-ME: NADP^+^-dependent malic enzyme, MDH: malate dehydrogenase, PPDK: pyruvate orthophosphate dikinase.

The expression of 12 genes related to photosynthesis, including 5 genes involved in chlorophyll synthesis (*BdUROD*, *BdCPO*, *BdProtox*, *BdChlH* and *BdCHLG*), 3 in photosynthetic electron transport (*BdLHCII*, *BdISP* and *BdFNR*) and 4 in CO_2_ assimilation (*BdPEPC*, *BdNADP-ME*, *BdMDH* and *BdPPDK*), were analyzed in buffalograss seedlings in response to 50 and 200 mM NaCl treatments for 6 h. As shown in Figure 7, after a 50 mM NaCl treatment for 6 h, all genes tested were upregulated, and except for *BdCPO*, *BdPEPC* and *BdMDH*, the relative expression levels of the other 9 genes at 50 mM NaCl were more than two-fold higher than those under the control condition (salt treatment for 0 h). *BdProtox*, *BdLHCII* and *BdNADP-ME* were even increased by over ten-fold (Figure 7, Supplementary Table S2). When buffalograss seedlings were exposed to high salinity (200 mM NaCl), the expression levels of *BdLHCII*, *BdISP* and *BdFNR* were continuously upregulated; meanwhile, the expression of *BdNADP-ME*, *BdMDH*, *BdPEPC* and *BdPPDK* involved in C_4_ carbon fixation remained at the control level. However, the expression levels of *BdProtox* and *BdChlH* significantly declined by 200 mM NaCl (Figure 7).

### 2.6. Effects of Salinity on Expression of Aquaporin Genes in Buffalograss

**Figure 8:**
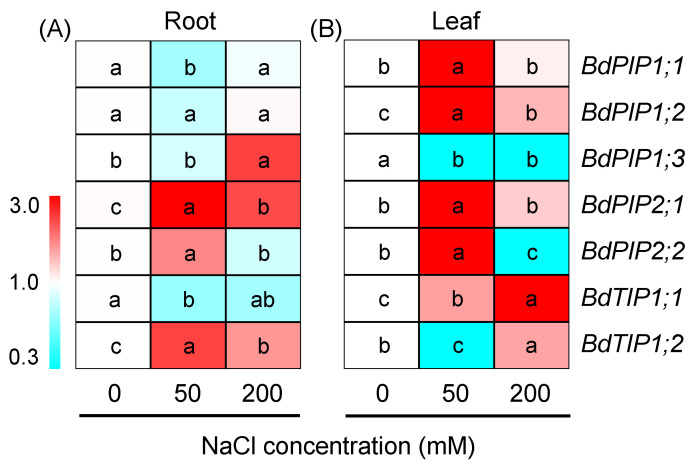
The expression of aquaporin genes in the roots (**A**) and leaves (**B**) of *B. dactyloides* under 0, 50 and 200 mM NaCl for 6 h. Expression of the *B. dactyloides ACTIN* gene was used as an internal control for normalization. Different lowercase letters within each gene indicate significant differences (*p* < 0.05, Duncan test). *PIP*: plasma membrane intrinsic protein, *TIP*: tonoplast intrinsic protein.

Buffalograss could significantly improve plant root activity to enhance water acquisition and simultaneously maintain the leaf water balance to adapt to saline environments (Figure 4). Therefore, the expression pattern of seven aquaporin genes (AQPs, including five plasma membrane-located *PIPs* and two tonoplast-located *TIPs*) was analyzed. In roots, the expression levels of three *PIP1s* (*BdPIP1;1 BdPIP1;2* and *BdPIP1;3*) were not induced by 50 mM NaCl and even showed slight downregulation, while two *PIP2s* (*BdPIP2;1* and *BdPIP2;2*) were significantly upregulated by 50 mM NaCl. After 200 mM NaCl treatment, the expression levels of *BdPIP1;3* and *BdPIP2;1* were upregulated two-fold more than those at control conditions (Figure 8A, Supplementary Table S3). Furthermore, the expression of *BdTIP1;2* in roots was upregulated under both 50 and 200 mM NaCl treatments (Figure 8A). In leaves, almost all tested *AQPs* (except for *BdPIP1;3*) were upregulated in response to 50 and/or 200 mM NaCl (Figure 8B). Among them, the expression of *BdPIP2;1* increased in both tissues after 50 and 200 mM NaCl treatments; its expression levels in the roots and leaves after 50 mM NaCl were 3.5- and 4.2-fold higher than those under the control condition, respectively (Figure 8, Supplementary Table S3).

### 2.7. Effects of Salinity on Expression of Genes Related to Na^+^ and K^+^ Transport in Buffalograss

**Figure 9:**
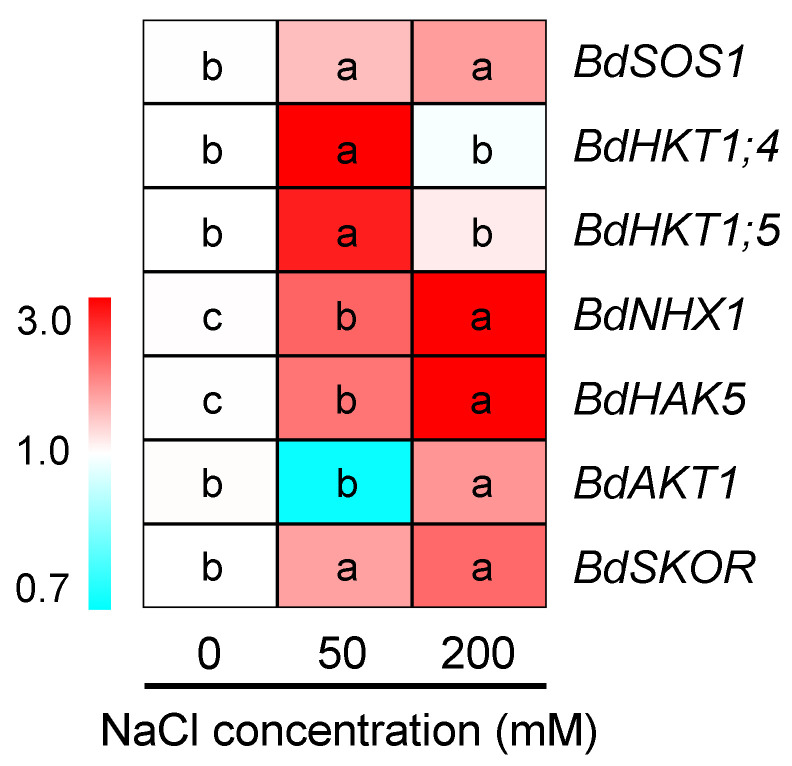
The expression of genes related to Na^+^ and K^+^ transport in the roots of *B. dactyloides* under 0, 50 and 200 mM NaCl for 6 h. Expression of the *B. dactyloides ACTIN* gene was used as an internal control for normalization. Different lowercase letters within each gene indicate significant differences (*p* < 0.05, Duncan test). SOS1: plasma membrane Na^+^/H^+^ antiporter, HKTs and HAK5: high-affinity K^+^ transporter, NHX1: tonoplast Na^+^/H^+^ antiporter, AKT1: inwardly rectifying K^+^ channel, SKOR: stelar K^+^ outwardly rectifying channel.

To understand how buffalograss seedlings maintained K^+^/Na^+^ homeostasis under saline conditions (Figure 5 and Figure 6), we further analyzed the expression of key genes related to Na^+^ and K^+^ transport in roots in response to 50 and 200 mM NaCl treatments for 6 h. As shown in Figure 9 and Supplementary Table S4, when plants were exposed to moderate salinity (50 mM NaCl), except for the inwardly rectifying K^+^ channel gene *BdAKT1*, other tested genes, encoding Na^+^/H^+^ antiporters (*BdSOS1* and *BdNHX1*), Na^+^-selective transporters (*BdHKT1;4* and *BdHKT1;5*) and K^+^ transporter/channel (*BdHAK5* and *BdSKOR*) were all upregulated (Figure 9). Under high salinity (200 mM NaCl), except for two *BdHKT1s* with no expression change, all the above genes were upregulated. In particular, the expression levels of *BdNHX1* and *BdHAK5* were 4.0- and 7.8-fold higher than those in control conditions, respectively (Figure 9, Supplementary Table S4).

## 3. Discussion

### 3.1. Buffalograss Maintains High Photosynthetic Capacity under Moderate Salinity by Stimulating the Expression of Photosynthesis-Related Genes

Salinity is generally harmful to the growth of most glycophytic species, but some special halophytes and xerophytes, such as *Suaeda* and *Atriplex* spp., can grow better at moderate salinity (within a 100 mM NaCl concentration) [41,42]. In the present work, under 50 and 100 mM NaCl treatments, the growth of buffalograss seedlings remained unaffected, and plant biomass, tillering ability and leaf relative membrane permeability were all comparable to plants under the control condition (Figure 1). Moreover, the root biomass was significantly increased under 50 and 100 mM NaCl treatments (Figure 1B,C). Differently, it has been reported that the growth of many glycophytes, especially traditional crops and turfgrasses in Poaceae such as wheat, rice, ryegrass and tall fescue, are inhibited by NaCl treatments lower than 100 mM [43,44,45]. Furthermore, although the leaf relative membrane permeability of buffalograss was significantly increased under the 200 mM NaCl treatment, the tissue biomass and tiller number were maintained at the same levels as those under the control condition (Figure 1), suggesting that buffalograss possesses a strong salt tolerance.

Biomass accumulation in higher plants directly depends on the photosynthetic capacity, while salinity reduced the photosynthetic capacity in most plants, resulting in a lower productivity [46,47]. The maintenance of a stable content of chlorophyll (Chl) is one of the most crucial physiological traits involved in the salt tolerance of plants, as it is directly linked to leaf photosynthetic capacity [48]. In this study, 50 and 100 mM NaCl had no obviously negative effect on the contents of Chl a and Chl b of buffalograss (Figure 2). Meanwhile, the Pn, Tr, Gs, Ci and WUE of buffalograss under 50 and 100 mM NaCl treatments were all maintained at the control levels (Figure 3). It was noticed that 200 and 400 mM NaCl treatments significantly decreased Chl contents and the Pn of buffalograss, which indicated that the external NaCl concentrations exceeding 200 mM would hamper the photosynthesis of this plant species.

It is known that salt stress influences Chl biosynthesis by restricting the expression levels of genes encoding relevant enzymes [44]. However, our results showed that moderate salinity (50 mM NaCl) stimulated the expression of genes associated with Chl biosynthesis in buffalograss, including four important rate-limiting enzymes, uroporphyrinogen decarboxylase (UROD), coproporphyrinogen-III oxidase (CPO), protoporphyrinogen oxidase (Protox) and magnesium chelatase H subunit (ChlH), involved in tetrapyrrole metabolism for the synthesis of Chl precursors [44,49,50], and a key enzyme (chlorophyll synthase, CHLG) catalyzing the last step of the Chl biosynthetic pathway [51] (Figure 7). Under high salinity (200 mM NaCl), the expressions of *BdProtox* (catalyzing the oxidation of protoporphyrinogen IX to protoporphyrin IX, the last enzyme of the common branch of the chlorophyll- and heme-synthesis pathway) [52] and *BdChlH* (involved in catalyzing the conversion of protoporphyrin IX to Mg-protoporphyrin IX in the chlorophyll pathway of tetrapyrrole metabolism) [50] in buffalograss were downregulated (Figure 7), which might be one of the crucial reasons why high salinity caused the reduction of Chl contents in buffalograss seedlings (Figure 2).

The expression of the *BdLHCII* gene coding an LHCII type I chlorophyll a-b binding protein (associated with PS II) was sharply upregulated by 11.9- and 24.9-fold under 50 and 200 mM NaCl, respectively (Figure 7, Supplementary Table S2), indicating that salinity might stimulate light energy harvest and transfer in the PS II complex of buffalograss. Additionally, 50 and 200 mM NaCl could upregulate the expression of *BdISP* (an iron-sulfur protein in the cytochrome b6/f complex) and *BdFNR* (chloroplast-targeted ferredoxin-NADP^+^ reductase associated with PS I) to promote electron transfer between PS II and PS I (Figure 7) [53,54].

The CO_2_ assimilation is the final stage in the oxygenic photosynthesis of higher plants, and C_4_ plants share stronger CO_2_ fixation capacity compared with C_3_ plants based on a CO_2_-concentrating mechanism [55]. Our study found that the expression levels of key enzymes in the C_4_ pathway of buffalograss were upregulated by 50 mM NaCl, including phosphoenolpyruvate carboxylase (PEPC), NADP-dependent malic enzyme (NADP-ME), malate dehydrogenase (MDH) and pyruvate orthophosphate dikinase (PPDK), which play critical roles in providing sufficient Ci and, thus, improving the Pn of buffalograss seedlings to adapt to salinity (Figure 3A,D). Thus, these results indicate that buffalograss can maintain high Chl synthesis and photosynthetic capacity under moderate salinity by stimulating the expression of photosynthesis-related genes, which was positively correlated with the constant biomass of buffalograss plants.

### 3.2. Efficient Water Uptake and Transport Capacity Plays an Important Role in the Salt Tolerance of Buffalograss

Stable water status is essential for plants to adapt to saline environments [17]. In the present study, the leaf RWC and WSD of buffalograss seedlings was unaffected by 50 and 100 mM NaCl (Figure 4A,B), suggesting that this species was able to maintain the water balance of photosynthetic organs that might result in the healthy growth of plants under moderate salinity. Unexpectedly, the root activity of buffalograss seedlings sharply increased after salt treatments (except for 400 mM NaCl) (Figure 4C). Combined with the appearance of increased root biomass after salt treatments (Figure 1B,C), these results suggest that buffalograss possesses high water acquisition and retention capacities under saline conditions, which is conducive to promote mineral nutrient absorption, and provide adequate raw material for photosynthesis.

Aquaporins (AQPs) can effectively regulate water flow through the plant tissues and maintain cellular water homeostasis by mediating the rapid transmembrane transport of water [18]. Plasma membrane-located *PIPs* are involved in regulating most of the water absorption by roots and water transportation across cells in various tissues, whereas tonoplast-located *TIPs* are extremely vital for cellular osmoregulation because they mediate the water exchange between the vacuole and cytosol [56,57]. *PIPs* in plants are further categorized into *PIP1* and *PIP2* sub-groups [57]. In this study, an interesting phenomenon was found that in buffalograss roots, the expression levels of three *PIP1* genes (*BdPIP1;1 BdPIP1;2* and *BdPIP1;3*) all showed a slight downregulated tendency under 50 mM NaCl (Figure 8A). It has been established that the downregulation of *PIPs* in roots facilitates cellular water conservation by reducing the water permeability of the root cell membrane [58,59]. Given that the hydration status of buffalograss under 50 mM NaCl treatment was unaffected (Figure 4A), the downregulation of *BdPIP1;1*, *BdPIP1;2* and *BdPIP1;3* might also be involved in the root water conservation of buffalograss. In contrast, the expressions of the tested *PIP2* genes (*BdPIP2;1* and *BdPIP2;2*) in roots were upregulated after 50 mM NaCl treatment (Figure 8A), which were likely involved in water uptake to sustain plant water homeostasis at moderate salinity. Under 200 mM NaCl, the expression abundances of *BdPIP1;3* and *BdPIP2;1* in roots were upregulated (Figure 8A). Previous studies found that *PIP1;3* and *PIP2;1* are highly expressed in the epidermal cells of roots under salt and drought stresses [60,61]. Therefore, the upregulation of *BdPIP1;3* and *BdPIP2;1* under 50 and/or 200 mM NaCl treatments might be conducive to the water absorption of buffalograss roots and, hence, enhance root activity.

The *TIP* subfamily in plants was divided into five sub-groups (*TIP1-5*) [62], and *TIP1s* were found in the lytic vacuole membrane and abundantly expressed in salt-treated roots to regulate water transport [63,64]. In buffalograss roots, the expression of *BdTIP1;2* was upregulated by 50 and 200 mM NaCl treatments (Figure 8A), which may be involved in water accumulation in root cell vacuoles to improve water retention capacity. Furthermore, a study found that *AtTIP1;2* facilitates the emergence of new lateral root primordia [65]. We speculate that *BdTIP1;2* may have similar functions to stimulate lateral root development and thereby contribute to a higher root biomass and root activity in buffalograss plants after salt treatments (Figure 1B,C and Figure 4C).

In contrast, in buffalograss leaves, almost all tested aquaporin genes (except for *BdPIP1;3*) were upregulated in response to 50 and/or 200 mM NaCl (Figure 8B), suggesting that there was also a well-developed water transport system in buffalograss leaves to maintain leaf water homeostasis under saline environments. Meanwhile, *PIPs* can also transport CO_2_ and are involve in CO_2_ diffusion across the plasma membrane of mesophyll cells [66,67]; we speculate that the upregulation of *PIPs* in buffalograss leaves under NaCl treatments would increase the membrane permeability for CO_2_ diffusion to contribute to maintaining a high Pn.

### 3.3. Maintaining K^+^/Na^+^ Homeostasis Is a Crucial Strategy for Buffalograss to Adapt to Saline Environments

Maintaining constant intracellular K^+^ and Na^+^ homeostasis is crucial for plants adapting to saline environments [12,68], which is mainly reflected in two aspects. One is the reduction in Na^+^ accumulation in the cytoplasm of photosynthetic organs, another is the improvement in K^+^ retention in the leaf mesophyll. In our study, the Na^+^ concentration in the roots of buffalograss was higher than that in the stems and leaves (Figure 5A–C), indicative of the strong leaf Na^+^ exclusion ability of buffalograss under salt stress. For most glycophytes, salinity stress seriously affects plant K^+^ acquisition and the accumulation of K^+^ in shoots [69,70,71]. Surprisingly, the K^+^ concentrations were maintained in stems, and even significantly increased in the leaves of buffalograss under NaCl treatments (Figure 5E,F). In addition, 50 mM NaCl had no effect on leaf K^+^/Na^+^ ratio in buffalograss, and this parameter was far more than 1 (it is generally thought that leaf K^+^/Na^+^ < 1 will severely inhibit plant growth [27]) under 100–400 mM NaCl treatments (Figure 6C). These results suggested that buffalograss possesses an atypical ability to maintain K^+^ and Na^+^ homeostasis under salt stress.

To obtain the optimal K^+^/Na^+^ ratio under saline stress, K^+^ absorption and K^+^ transport from the roots to shoots through the xylem are required to be enhanced, and Na^+^ influx and transport to the shoots should be limited [32,68]. Several proteins involved in root Na^+^ transport, such as SOS1 and HKTs, play vital roles in restricting Na^+^ overaccumulation in shoots under salt stress [29,72,73]. Our results showed that the expression of *BdSOS1* was upregulated in roots under two salinity levels (50 and 200 mM NaCl) (Figure 9), which should contribute to promote Na^+^ exclusion from the root tip cells [29]. We also observed that two HKT1 genes (*BdHKT1;4* and *BdHKT1;5*) were upregulated by 50 mM NaCl (Figure 9). Previous studies demonstrated that *HKT1;4* and *HKT1;5* are highly expressed in roots under salt stress involved in Na^+^ retrieval from the xylem into xylem parenchyma cells to limit excessive Na^+^ accumulation in shoots [72,73,74], suggesting that BdHKT1;4 and BdHKT1;5 in buffalograss roots might play similar roles. The upregulation of *BdSOS1* and two *BdHKT1s* in roots by 50 mM NaCl is conducive to the leaf Na^+^ exclusion of buffalograss, which closely correlated with holding the lower leaf/root Na^+^ ratio at moderate salinity. Inversely, the lack of change in the expression of *BdHKT1s* at 200 mM NaCl may be one of the important reasons for the increase in leaf/root Na^+^ ratio in leaves under high salinity. Additionally, the expression levels of another gene encoding for NHX1 protein function as the tonoplast Na^+^/H^+^ antiporter were increased 1.2- and 4.0-fold under 50 and 200 mM NaCl treatments for 6 h, respectively (Figure 6, Supplementary Table S4), which are capable of compartmentalizing more Na^+^ into root vacuoles, thereby improving the osmotic adjustment ability to enhance water uptake and reducing Na^+^ toxicity for root cells [75,76].

K^+^ uptake capacity in the high-affinity range of concentrations is a prerequisite for plants to sustain K^+^ homeostasis under saline environments, and HAK5 (high-affinity K^+^ transporter) and AKT1 (inwardly rectifying K^+^ channel) in plants have been shown to be the main proteins involved in this process [77,78]. The results of the present study showed that the expression of *BdHAK5* in buffalograss roots was significantly increased by two salinity; specifically it was 7.8-fold higher than that in control conditions at 200 mM NaCl (Figure 9, Supplementary Table S4). The expression of *BdAKT1* was slightly downregulated by 50 mM NaCl, whereas it was upregulated by 200 mM NaCl (Figure 9). These results suggest that *BdHAK5* in buffalograss may play a major role in root K^+^ acquisition in the presence of moderate salinity, and that *BdAKT1* is primarily involved in K^+^ uptake under high salinity. When K^+^ absorbed by plant root epidermal cells is transported to the root xylem through the apoplast and symplast pathways, it needs to be loaded into xylem sap for translocation from roots to shoots, and SKOR (stelar K^+^ outwardly rectifying channel) was a key protein reported to mediate the K^+^ loading process [32]. In our study, *BdSKOR* expression levels in roots were increased by two salinity, and this upregulation enhanced K^+^ loading into the xylem, which correlated with K^+^ accumulation in the stems and leaves of buffalograss observed in the present results. In consequence, the increased expression of *BdHAK5* and *BdSKOR* as well as that of *BdSOS1* and *BdHKT1s* in buffalograss roots when plants were exposed to moderate salinity (50 mM NaCl) can account for the higher K^+^/Na^+^ ratio in leaves (Figure 6C).

## 4. Materials and Methods

### 4.1. Plant Growth Conditions and Salt Treatments

The seeds of buffalograss (*Buchloe dactyloides* (Nutt.) Engelm.) were obtained from Beijing Clover Ecotechnology Co., LTD., Beijing, China, in 2021. Considering the naturally low germination rate of buffalograss seeds, they were first sown in the experimental field of Northwest A&F University, and then the original stolon sections were excised from the middle of healthy buffalograss plants by using sharp scissors and their bases were placed into black plastic pots (10 cm high × 8 diameter, 4 cuttings/pot) filled with sterilized quartz sand (5–8 mm particle size). The pots were transferred into a greenhouse with a temperature of 28 °C/25 °C (day/night), a photoperiod of 16/8 h (light/dark, the flux density was about 800 μmol/m^2^/s) and a relative humidity of approximately 65%. When the cuttings generated adventitious roots (about 10 days), uniform seedlings were chosen for further culture and irrigated with modified Hoagland nutrient solution [79].

After five weeks of preculture, buffalograss seedlings were treated with Hoagland nutrient solution containing additional 0 (control), 50, 100, 200 or 400 mM NaCl for 8 days, and 100, 200 and 400 mM NaCl treatment solutions were supplied incrementally by 50 mM each day to avoid salinity shock until the final concentrations were achieved. The treatment solutions were renewed every two days to keep a constant NaCl concentration. After 8 d, the plants were harvested for biomass measurement and physiological analysis, and six replicate seedlings (*n* = 6) were used for all measurements.

### 4.2. Measurement of Parameters Related to Growth

Tiller number was first counted. Then, the roots and shoots of individual seedlings were carefully separated, and tissue fresh weight (FW) was immediately measured after blotting off the surface water with filter paper. After that, all samples were oven-dried at 80 °C for 72 h to obtain dry weight (DW).

The leaf relative membrane permeability (RMP) was assessed according to the method described by Gibon et al. [80]. About 0.5 g of fresh mature leaves was cut into pieces and put in deionized water. After vacuuming for 30 min (10 min × 3 times) until the leaves were completely sunk, the leaf samples were shaken gently at 25 °C for 1.5 h, and then the initial electrolyte leakage (E1) was measured by using a conductivity meter (EC215, Hanna Instruments, Padovana, Italy). Finally, the leaf samples were incubated in a boiling water bath for 15 min, and the total electrolyte leakage (E2) was recorded after cooling. The RMP (%) was calculated as (E1/E2) × 100.

### 4.3. Measurement of Parameters for Photosynthesis Relations

The contents of chlorophylls were determined using fresh leaves according to the method described by Lichtenthaler [81]. Briefly, 0.1 g fresh leaf samples were cut into pieces and then immediately soaked in 10 mL of extraction solution, which was composed of 80% acetone and 95% ethyl alcohol at a volume ratio of 1:1. After extracting for 24 h in the dark, the supernatant was collected via centrifugation. Then, the absorbance at 645 nm and 663 nm was recorded using a UV spectrophotometer (UV-2102C, Unico Instrument Co., Ltd., Shanghai, China). The contents of chlorophyll *a* (Chl a), chlorophyll *b* (Chl b) and total chlorophyll (total Chl) were calculated by using the equations described by Arnon [82].

The leaf net photosynthesis rate (Pn), transpiration rate (Tr), stomatal conductance (Gs) and intercellular CO_2_ concentration (Ci) were measured in the greenhouse between 3 h and 5.5 h after the start of the photoperiod using a portable photosynthesis system (LI-6400XT, LI-COR, Lincoln, NE, USA). The intrinsic water use efficiency (WUE) was calculated as Pn/Gs [83]. Leaf areas were estimated using an Epson Perfection 4870 photo-scanner (Epson America, Inc., Long Beach, CA, USA).

### 4.4. Measurement of Parameters for Root Activity and Leaf Water Status

The root activity was determined using fresh roots according to the tetrazolium (TTC) staining method as described by Zhang et al. [84]. About 0.5 g of fresh tender roots was immersed in 10 mL of mixed solution of 0.4% TTC and phosphate buffer. After incubating at 37 °C in the dark for 3 h, 2 mL 1 M H_2_SO_4_ was immediately added to terminate the reaction. Subsequently, the root samples were dried with filter paper and then extracted with 10 mL of methyl alcohol. After being sealed in the dark for 3 h until the roots turned white, the absorbance of red extractant was recorded at 485 nm using a UV spectrophotometer. Root activity was calculated using the following formula: root activity (mg/g FW/h) = amount of TTC reduction (mg)/fresh root weight (g) × time (h).

The leaves were excised from buffalograss seedlings and the FW was weighed immediately. Then, the turgid weight (TW) was determined after soaking the leaves in distilled water at 4 °C overnight. Finally, the leaves were oven-dried at 80 °C for 72 h to obtain the DW. The leaf relative water content (RWC) and water saturation deficit (WSD) were calculated according to the following formulas: RWC (%) = 100 × (FW − DW)/(TW − DW); WSD (%) = 100 × (TW − FW)/(TW − DW) as described by Chen et al. [28].

### 4.5. Measurement of Na^+^ and K^+^ Concentrations in Tissues

The K^+^ and Na^+^ concentrations in tissues were measured according to the methods of Pan et al. [17]. Firstly, the roots, stems and leaves of seedlings were separated, and then the roots were rinsed with distilled water and placed in ice-cold 20 mM LiNO_3_ solution for 8 min to exchange cell-wall-bound salts. After oven drying at 80 °C for 72 h, the K^+^ and Na^+^ were extracted from the dried tissues using 100 mM acetic acid at 90 °C for 2 h, and the K^+^ and Na^+^ concentrations were then determined with a flame spectrophotometer (Model 410, Sherwood Scientific, Ltd., Cambridge, UK).

### 4.6. RNA Extraction and cDNA Synthesis

According to the results of physiological indicators, buffalograss could adapt well to 50 mM NaCl and this salt concentration could even stimulate its growth, while 200 mM NaCl should be the critical concentration for this species to suffer from salt stress. Therefore, 50 and 200 mM NaCl were chosen as salt treatment concentrations in the gene expression analysis. Four-week-old buffalograss seedlings were treated with Hoagland nutrient solution supplemented with 0 (control), 50 or 200 mM NaCl. After treatments for 6 h, the roots and leaves of seedlings were collected, immediately frozen in liquid nitrogen and stored at −80 °C. After the frozen roots and leaves were ground into powder, the total RNA was extracted with an E.Z.N.ATM plant RNA kit (Omega Bio-Tek, Norcross, GA, USA). The integrity and purity of RNA were checked using 1% agarose gel electrophoresis and spectrophotometric analysis using a NanoDrop 2000 (Thermo Scientific, Pittsburgh, PA, USA), and then the first-strand cDNA was synthesized from 2 μg of total RNA using a PrimeScript™ RT Master Mix with gDNA eraser (Perfect Real Time) kit (TaKaRa, Biotech Co., Ltd., Dalian, China).

### 4.7. Gene Expression Analysis

We chose 12, 8 and 7 genes related to photosynthesis, aquaporin and ion transport, shown in Supplementary Table S1, respectively, to analyze their expression levels in response to different NaCl treatments. Quantitative real-time PCR (qRT-PCR) was used to estimate the expression levels of the genes. The primers used in qRT-PCR were designed with Primer 5.0 (Premier Biosoft International, Palo Alto, CA, USA) and are listed in Supplementary Table S1. Buffalograss *BdACTIN* (beta actin) was used as the reference gene for RNA normalization. SYBR Green Real-Time PCR Master Mix (Takara, Biotech Co., Ltd., Dalian, China) was used for qRT-PCR on a QuantStudio^®^5 real-time PCR system (Life Technologies, Waltham, MA, USA). Three biologically independent replicates were used to obtain the gene expression quantification data. All PCR reactions were performed with three replicates, and 3 μL first-strand cDNA after ten times dilution was used for each of the qRT-PCR reactions with a 20 μL reaction system. Finally, the relative expression levels of genes were calculated using the 2^−ΔΔCt^ method according to Duan et al. [85].

### 4.8. Statistical Analysis

SPSS 19.0 statistical software (SPSS Inc., Chicago, IL, USA) was used for statistical analyses. Data were subjected to a one-way ANOVA (Duncan’s test, *p* < 0.05). Figure 1, Figure 2, Figure 3, Figure 4, Figure 5 and Figure 6 were drawn with SigmaPlot 14.0 (Systat Software Inc., San Jose, CA, USA). All data in the physiological experiments are presented as mean ± SE (*n* = 6). Figure 7, Figure 8 and Figure 9 were drawn using the MEV (Multiple Experiment Viewer) software, and the qRT-PCR data are presented in Supplementary Tables S2–S4 as mean ± SE (*n* = 3).

## 5. Conclusions

In conclusion, buffalograss possesses a prominent salt tolerance, as its growth, photosynthesis and water status were unaffected when external NaCl concentration was up to 200 mM. Moreover, this species could efficiently restrict Na^+^ overaccumulation and increase K^+^ concentration in leaves to maintain a high K^+^/Na^+^ ratio under salt stress. After NaCl treatments, buffalograss could maintain high leaf photosynthetic capacity and water balance by upregulating the expression of many genes associated with chlorophyll biosynthesis, photosynthetic electron transport, CO_2_ assimilation as well as *PIP* and *TIP* aquaporins encoding genes. In addition, several genes related to Na^+^ transport such as *BdSOS1*, *BdHKT1;4*, *BdHKT1;5* and *BdNHX1*, and genes related to K^+^ transport such as *BdHAK5* and *BdSKOR*, should play key roles in the maintenance of K^+^/Na^+^ homeostasis in buffalograss leaves under salt stress. Further studies on the function of those genes would help to elucidate the salt tolerance mechanisms employed by buffalograss, and, thus, provide a theoretical basis for the cultivation of this species in salinized areas.

## Data Availability

The data that support the findings of this study are available on request from the corresponding author.

## Supplementary Materials

The following supporting information can be downloaded at: https://www.mdpi.com/article/10.3390/plants12132459/s1. Table S1: The primers for qRT-PCR analysis; Table S2: The relative expression level of genes related to photosynthesis in *B. dactyloides* under 0 (Control), 50 and 200 mM NaCl for 6 h using qRT-PCR; Table S3: The relative expression level of aquaporin genes in the roots and leaves of *B. dactyloides* under 0 (Control), 50 and 200 mM NaCl for 6 h using qRT-PCR; Table S4: The relative expression level of genes related to Na^+^ and K^+^ transport in the roots of *B. dactyloides* under 0 (Control), 50 and 200 mM NaCl for 6 h using qRT-PCR.
