# Supplementary material for: Photosynthesis, Water Status and K+/Na+ Homeostasis of Buchoe dactyloides Responding to Salinity
Source: Plants (Basel). 2023 Jun 27;12(13):2459. doi: 10.3390/plants12132459 (PMC10346696; doi:10.3390/plants12132459)
Supplement: Supplementary file 1 [file plants-12-02459-s001.zip › plants-2447000-supplementary.pdf]

Table S1 The primers for qRT-PCR analysis.

Genes	Forward primers (5′-3′)	Reserve primers (5′-3′)
**Photosynthesis-related genes**		
BdUROD	TGTCCAACATTACCAGCCTTCT	GATTGGGGTCTAACATAGCAGTTC
BdCPO	CAGTACCACCACCAAACCACC	GCTTCATCCCAAGAACCCAT
BdProtox	TACGTCCTCAGATGCCTCCAC	AAGGTGCTCGTGTCCGTGTC
BdChlH	TTGAGCACGGAGTAGATGGAGT	GAGGAAAACTAAGGAGGAGAAGCG
BdCHLG	TGACATCATACTTCACAGGGTCC	GGCACTGCTTGGTCTAACAATTC
BdLHCII	TCCTTGACCTTGAGCTCCG	ATGGGATTCGTCGAGGGCTAC
BdISP	GTCCCCTACGGCTCCTTCTT	CCACTCGTCCACCTTGATGT
BdFNR	CTAGTAGACCTCCACATGCCATT	GATTATGCCCGCGATCCAG
BdPEPC	CTCCTCGACAGTGTTAAAGATTGC	AGCCCTACCGTATCGTGCTT
BdNADP-ME	ATGCTCCCGTACTTCTGGC	TTCTACAAGCTGCTCATCGACC
BdMDH	GCGCTCTTGATTTCAGGCTG	CCTTCAGTTCATCGGTTCCTTGT
BdPPDK	GTCCGGCATAATCCAATTCC	GCTCAAGGTCCTGGCTAACG
**Aquaporins**		
BdPIP1;1	CAATCCGCACCACAACCATT	ATCCTCCTCCTTGCCCTCCAT
BdPIP1;2	GGATGAAGGTGCCGACGAT	GTCAAAGGATTCCAGCAGACG
BdPIP1;3	CCCACCCAGAAAATCCAATG	CAACCATCCCCATCACAGG
BdPIP2;1	ATGGATCTTCTGGGTTGGACC	GTTGCTCCTGAATGACCCG
BdPIP2;2	AACATCCAGTGATCCTCCCAT	GTTTCGCGGTGTTCATGGT
BdTIP1;1	AAGGTCACGGCAGGGTTCA	ACGCCTTTGGCCTGTTCGT
BdTIP1;2	GATCGTACCCAAGCTACCCTTC	CTGGTGTTGGAGATCGTGATG
**Ion transports**		
BdSOS1	AATGAAGTGCGGTGCCTGAG	GCAATGGAGGAAGAAAACAACTC
BdHKT1;4	GGCCCTGTTTATGGTTTCCTT	GTCCTCACCGTGCTGATGTT
BdHKT1;5	GTTGCTGATCTTGCTCCCGT	CTACCACCTTACACCACATTTCTG
BdNHX1	GCATAAGACCAGCCCACCAT	CTGCTTTTGTTTTCCCACTATCC
BdHAK5	ATGCAATGGAGCCTTGTAGTG	AGTTGTACCTCACTTACGCATCC
BdAKT1	CGATTGGAGAAAGATAGGCACTT	CGGCAAGAAGATAGTAGAAGCATG
BdSKOR	ACGCAAAGAGGAGGTGAGGT	CTCCAGGTGCCAGAAGAAGTC
**Reference gene**		
BdACTIN	TCTGGATCTTGCTGGGCGT	TGCGAGCTTCTCCTTGATGT

Table S2 The relative expression level of genes related to photosynthesis in *B. dactyloides* under 0 (Control), 50 and 200 mM NaCl for 6 h using qRT-PCR. Data are means (± SE), *n* = 3. Different letters (a, b, c) indicate significant differences at $P < 0.05$ (Duncan test).

Genes	Control	50 mM NaCl	200 mM NaCl	Encoding proteins
BdUROD	1.02 ± 0.12b	3.17 ± 0.14a	1.13 ± 0.08b	Uroporphyrinogen decarboxylase
BdCPO	1.01 ± 0.08b	2.15 ± 0.12a	0.99 ± 0.17b	Coproporphyrinogen-III oxidase
BdProtox	1.11 ± 0.23b	18.81 ± 1.21a	0.70 ± 0.16c	Protoporphyrinogen oxidase
BdChlH	1.02 ± 0.10b	3.21 ± 0.34a	0.49 ± 0.05c	Magnesium chelatase H subunit
BdCHLG	1.03 ± 0.18b	5.34 ± 0.17a	1.27 ± 0.11b	Chlorophyll synthase
BdLHCII	1.05 ± 0.18c	13.50 ± 2.31b	27.16 ± 2.52a	LHCII type I chlorophyll a-b binding protein
BdISP	1.05 ± 0.17c	6.23 ± 0.41a	2.49 ± 0.08b	Cytochrome b6/f complex iron-sulfur subunit
BdFNR	1.00 ± 0.01c	8.06 ± 0.40a	2.25 ± 0.12b	Ferrdoxin-$NADP^+$ reductase
BdPEPC	1.02 ± 0.12b	1.84 ± 0.03a	1.02 ± 0.02b	Phosphoenolpyruvate carboxylase
BdNADP-ME	1.14 ± 0.08b	15.89 ± 1.36a	1.26 ± 0.10b	$NADP^+$-dependent malic enzyme
BdMDH	1.02 ± 0.14b	2.07 ± 0.14a	1.13 ± 0.05b	Malate dehydrogenase
BdPPDK	1.04 ± 0.13b	5.91 ± 0.83a	1.57 ± 0.16b	Pyruvate orthophosphate dikinase

Table S3 The relative expression level of aquaporin genes in roots and leaves of *B. dactyloides* under 0 (Control), 50 and 200 mM NaCl for 6 h using qRT-PCR. Data are means (± SE), *n* = 3. Different letters (a, b, c) indicate significant differences at *P* < 0.05 (Duncan test).

**Genes**	**Roots**			**Leaves**		
	**Control**	**50 mM NaCl**	**200 mM NaCl**	**Control**	**50 mM NaCl**	**200 mM NaCl**
BdPIP1;1	1.03 ± 0.17a	0.75 ± 0.02b	0.97 ± 0.11a	1.00 ± 0.22b	5.75 ± 0.30a	1.13 ± 0.22b
BdPIP1;2	1.01 ± 0.11a	0.85 ± 0.06a	1.06 ± 0.16a	1.00 ± 0.04c	4.30 ± 0.44a	1.58 ± 0.08b
BdPIP1;3	1.00 ± 0.04b	0.88 ± 0.03b	2.49 ± 0.07a	1.00 ± 0.16a	0.06 ± 0.00b	0.08 ± 0.00b
BdPIP2;1	1.04 ± 0.02c	4.61 ± 0.46a	2.41 ± 0.12b	1.00 ± 0.10b	5.23 ± 0.31a	1.40 ± 0.25b
BdPIP2;2	1.01 ± 0.10b	1.94 ± 0.17a	0.87 ± 0.07b	1.00 ± 0.02b	5.78 ± 0.84a	0.31 ± 0.03c
BdTIP1;1	1.01 ± 0.10a	0.72 ± 0.01b	0.75 ± 0.08ab	1.00 ± 0.03c	1.75 ± 0.13b	9.66 ± 0.87a
BdTIP1;2	1.00 ± 0.05c	2.48 ± 0.05a	1.82 ± 0.07b	1.00 ± 0.30b	0.21 ± 0.01c	1.72 ± 0.06a

Note: The expression analysis of aquaporin genes was based on the root samples under control conditions. The expression level in leaves was uniformly convert to 1 in control group to calculate the expression level under salt treatments, so the relative expression level in leaves under the control was all shown as “1.00”.

Table S4 The relative expression level of genes related to Na^+ and K^+ transport in roots of *B. dactyloides* under 0 (Control), 50 and 200 mM NaCl for 6 h using qRT-PCR. Data are means (± SE), *n* = 3. Different letters (a, b, c) indicate significant differences at $P < 0.05$ (Duncan test).

Genes	Control	50 mM NaCl	200 mM NaCl	Encoding proteins
BdSOS1	1.01 ± 0.10b	1.51 ± 0.06a	1.77 ± 0.06a	Plasma membrane Na^+/H^+ antiporter
BdHKT1;4	1.00 ± 0.04b	4.11 ± 0.18a	0.99 ± 0.05b	High-affinity K^+ transporter
BdHKT1;5	1.00 ± 0.03b	2.77 ± 0.17a	1.16 ± 0.16b	High-affinity K^+ transporter
BdNHX1	1.02 ± 0.13c	2.22 ± 0.17b	5.02 ± 0.54a	Tonoplast Na^+/H^+ antiporter
BdHAK5	1.01 ± 0.12c	2.10 ± 0.11b	8.81 ± 1.28a	High-affinity K^+ transporter
BdAKT1	1.02 ± 0.15b	0.71 ± 0.04b	1.83 ± 0.19a	Inwardly rectifying K^+ channel
BdSKOR	1.01 ± 0.07b	1.74 ± 0.11a	2.18 ± 0.26a	Stelar K^+ outwardly rectifying channel
